# The relationship between Long Noncoding RNA (lncRNA) Small Nucleolar RNA Host Gene 12 (SNHG12) expression in solid malignant tumors and prognosis of tumor patients: A systematic review and meta-analysis

**DOI:** 10.1097/MD.0000000000022247

**Published:** 2020-10-09

**Authors:** Zixi Huang, Wen Zhuo, Ruoqing Xu, Zilong Wu, Ying Xiong, Zhenyan Xu

**Affiliations:** a Department of General Medicine; b Department of Cardiovascular Medicine, the Second Affiliated Hospital of Nanchang University, Nanchang, Jiangxi; c Tongji Medical College, Huazhong University of Science and Technology, Wuhan, Hubei; d The First Clinical Medical College, Nanchang University, Nanchang, Jiangxi, China.

**Keywords:** SNHG12, tumor, prognosis, meta-analysis

## Abstract

**Background:**

Small nucleolar RNA host gene 12 (SNHG12) has been demonstrated to be a long noncoding RNA (lncRNA) that facilitates the progression of several solid malignant tumors. However, whether the expression level of SNHG12 in solid malignant tumors is associated with patients prognosis have not been investigated.

**Methods:**

We systematically searched PubMed, EMBASE and Cochrane Library from Jan 1, 1950 to Mar 24, 2020 for randomized controlled trials published in English on SNHG12 expression in solid malignant tumors. We used the Newcastle-Ottawa Scale to assess the quality of articles. The HRs and 95%CI that extracted from Kaplan–Meier curves were used to perform the forest plot using a fixed-effects model. The meta-analysis was performed according to the Preferred Reporting Items for Systematic Reviews and Meta-Analyses (PRISMA) guidelines.

**Results:**

Thirteen articles containing 821 patients were included in this systematic review and meta-analysis. The result showed that high lncRNA SNHG12 expression is significantly associated with poor overall survival (OS) (HR = 1.94, 95% CI: 1.56–2.41, *P* < .001) and the studies are lack of statistically significant heterogeneity (*P*= .878, *I*^2^ = 0.0%). Beggs plot and Eggers test were applied to testify no publication bias existence in these studies. Subgroup analyses were performed and the result showed that TNM stage, lymph node metastasis and tumor type can influence the patients outcome, while there was no significantly correlation between SNHG12 expression and gender.

**Conclusions:**

The systematical review and meta-analysis synthetically analyzed 13 articles including 821 patients with ten types of solid malignant tumors, concluding that higher lncRNA SNHG12 expression is significantly associated with worse clinical prognosis.

## 1 Introduction

Cancer incidence and mortality are rapidly growing worldwide. Cancer is expected to rank as the leading cause of death in the 21st century. Rebecca et.al showed that 1,762,450 new cancer cases and 606,880 cancer deaths occurred in 2019 in United States.^[1]^

For decades, scientists spared no effort to seek a cure for cancer. Many treatment options exist, such as surgery, chemotherapy, radiation therapy, hormonal therapy, targeted therapy and palliative care. Among all the therapeutic methods, targeted cancer therapies are expected to be more effective than other forms of treatment by accurately interfering with oncocytes metastasis on the molecular level, with less harm to normal cells. Many targeted drugs aimed at specific targets have been brought out, such as Gefitinib, which targets the epidermal growth factor receptor tyrosine kinase, and Imatinib, a BCR-ABL tyrosine kinase inhibitor. However, the survival rate for most cancer remains low, which always presented as Overall Survival (OS) to estimate the patient's prognosis, and the relationship between many other biological target and cancers remains unknown. Hence, there remains many biomarkers for us to explore.

LncRNA (long non-coding RNAs) are a kind of transcripts that do not translate to protein, with length longer than 200 bp.^[2]^ As a member of noncoding RNAs group, lncRNA are functional elements that with a wide range of physiological and pathophysiological processes, which can be classified as signaling, decoy, guide, and scaffold lncRNAs.^[3]^ Several previous studies have confirmed the role lncRNA plays in plenty biological processes, such as regulating chromatin dynamics, gene expression, growth, differentiation, cell cycle, imprinting and splicing.^[4–6]^ LncRNA were found to participate in the occurrence and development of several disease, such as cardiovascular disease, nervous system disease and hematological system diseases.^[7]^ Previous studies have confirmed the role lncRNA plays in atherosclerosis, ischemic stroke^[8,9]^ and multiple myeloma.^[10]^ Moreover, lncRNA are in closely connection with tumorigenesis, which can be act as oncogenes or tumor suppressor genes. High lncRNA expression has been observed in tumor tissues compared to normal tissues, such as MALAT1^[11]^ and H19.^[12]^

Small nucleolar RNA host genes (SNHGs) are host genes for snoRNAs that could not code for protein. LncRNA small nucleolar RNA host genes are a member of the class of SNHGs, which were found to be involved in cancer progression and cell apoptosis.^[13]^ Long non-coding RNA small nucleolar RNA host gene 12 (SNHG12) is a novel lncRNA located in chromosome 1 and 675 nucleotides in size, which has been reported to be up-regulated in several tumor cells, such as osteosarcoma,^[14]^ nasopharyngeal carcinoma^[15]^ and endometrial carcinoma.^[16]^ Moreover, SNHG12 played important roles in cancer cell proliferation and migration. Recently, several studies have reported that high SNHG12 expression is in connection with low survival rate of cancer patients, including nasopharyngeal carcinoma,^[17]^ osteosarcoma,^[18]^ colorectal cancer,^[19,20]^ gastric carcinoma,^[21–23]^ hepatocellular carcinoma,^[24]^ glioma,^[25]^ non-small cell lung cancer,^[26]^ cervical cancer,^[27]^ esophageal squamous cell carcinoma^[28]^ and prostate cancer.^[29]^ However, most published research is limited by a low sample size, the prognostic value of expression of the lncRNA SNHG12 remains unclear. Therefore, we conduct this meta-analysis to explored whether the relationship exists between lncRNA SNHG12 expression and solid malignant tumor prognosis.

## 2 Metetial and methods

### 2.1 Data sources and search strategy

Two reviewers (H-ZX and X-RQ) independently selected relevant studies published between Jan 1, 1950 and Mar 24, 2020, by searching PubMed, EMBASE, Cochrane Library. The keywords used for searching are as follows: “Neoplasia”, “Neoplasm”, “Tumor”, “Cancer”, “Malignant Neoplasms”, “Benign Neoplasms”, “long non-coding RNA small nucleolar RNA host 12, human”, “SNHG12 lncRNA, human”, “SNHG12”.

**Figure 1:**
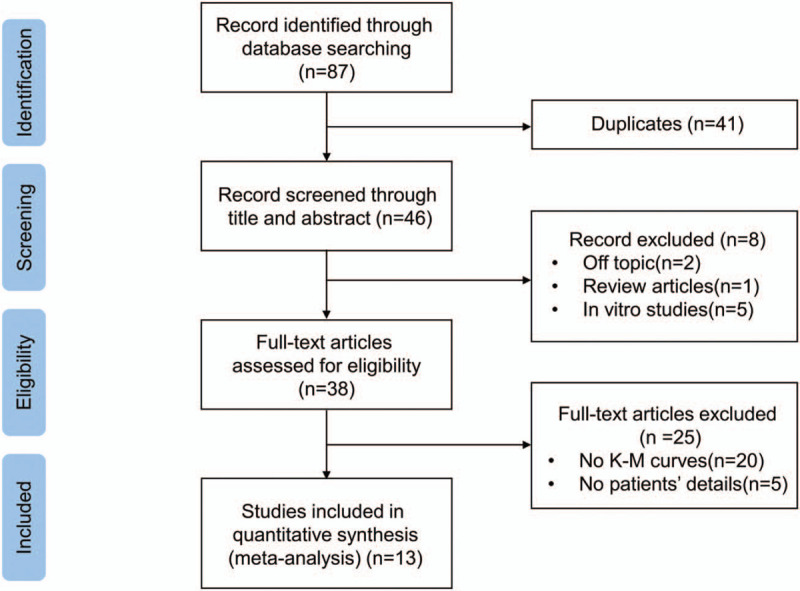
Flowchart of paper screening process.

The articles searched as was stated above were managed by Endnote X8. After the duplications were excluded, the title, abstract and full text were read separately by 2 reviewers (H-ZX and Z-W) to identify weather the studies are eligible to bring into analysis. We removed the studies that were unfit for the analysis and classified them by reasons. The details are showed in Fig. 1.

### 2.2 Eligibility criteria and exclusion criteria

Studies met the inclusion criterion if:

1. The cases were diagnosed as solid malignant tumors based on pathological results.
2. The sample size of patients was no less than 30.
3. The follow-up period was no less than 45 months.
4. qRT-PCR was used to quantify the expression levels of SNHG12.
5. The experiments were approved by the Medical Ethics Committee and informed consent was signed.
6. The patients did not undergo any preoperative anti-cancer therapy before.
7. Overall survival (OS) was used to evaluate the prognosis of patients and was performed in Kaplan-Meier curves.
8. The detailed information of patients was described, such as gender, age, tumor stage, tumor size and lymph node metastasis.

Studies were excluded if:

1. The articles were about in vitro or in vivo experimental studies.
2. The sample size of patients was less than 30.
3. The follow-up period did not reach 45 months.
4. The studies were lack of available clinical parameters.
5. The full text could not be found.
6. Kaplan–Meier curves were not used to describe the prognosis of patients.
7. The studies were case reports, letters, review articles, expert opinions, meeting records, commentaries, meta-analysis and clinical guidelines.

### 2.3 Data extraction

The data was extracted and the bias risk was assessed according to the PRISMA recommendation separately by 2 reviewers (H-ZX and Z-W) after the articles were filtrated. The information we obtained from each study are as follows: surname of the first author, publication place, publication date, total sample size, cancer type, sample source, tumor stage, lymph node metastasis, clinicopathological features, follow-up time, outcome measures, analysis type, and the detection method of SNHG12 expression. OS was used to evaluate the patients prognosis and were determined by Kaplan–Meier analysis. By using Engauge Digitizer 4.1 to analyzed the Kaplan–Meier survival curves, we converted the image into data. Hazard Ratio (HR) and 95% confidence intervals (95%CI) of each study were calculated by the HR calculations spreadsheet mentioned in Jayne F Tierneys article.^[30]^

### 2.4 Quality assessment

The Newcastle-Ottawa Scale (NOS) was used by 2 authors (H-ZX and Z-W) independently for assessing the quality of non-randomized studies. Quantitative evaluation was performed from selection, comparability and outcome. A star was provided for each question on the framework, with a star awarded when the article met the criterion and no star if the criterion was not met. Each paper could achieve a maximum star of 9 and the study considered to be of high value when it got more than 6 stars.

### 2.5 Statistical analysis

The HRs and 95%CI were calculated to perform forest plot using STATA12 and the heterogeneity was evaluated by Chi-Squared and *I*-squared test. Heterogeneity was existed when *P* value <.10 by Chi-Squared test. An *I*^2^ > 50% indicated substantial heterogeneity. When *I*^2^ < 50%, we consider the heterogeneity does not exist and the fixed-effects model may be used to pool the effect estimates in all meta-analyses. The funnel plot and the Galbraith plot were performed to evaluate the possibility of heterogeneity further. We researched the impact of single study to assess the study sensitivity. The Beggs funnel plot and Eggers test were used to assess the possibility of publication bias. To assess the heterogeneity further, subgroup analysis was performed. Statistical analyses were conducted using STATA12 (Stata Corp, College Station, TX, USA).

## 3 Results

### 3.1 Literature Screening

Fig. 1 shows the process of the document retrieval. 87 articles were retrieved using the search strategy which has been described in the section of the method, which were managed by Endnote X8. We found 44 articles in PubMed and 43 in EMBASE, while nothing was found in Cochrane Library. There were 46 articles left after duplicate checking. After glancing over the titles and abstracts, 38 articles were excluded (2 off topic, 1 review article and 5 in vitro studies). 25 articles were removed after intensive reading full articles because of incomplete data extraction (20 no K–M curves and 5 no patients details). Eventually, 13 articles were included in our final analysis. Interestingly, the including articles were both published by Chinese scholars and the patients were selected from Chinese hospitals.

### 3.2 Study Characteristics

Among the 13 included articles, all the cancer types are solid malignant tumor that verified by pathologic examination, including nasopharyngeal carcinoma,^[17]^ osteosarcoma,^[18]^ colorectal cancer,^[19,20]^ gastric carcinoma,^[21–23]^ hepatocellular carcinoma,^[24]^ glioma,^[25]^ non-small cell lung cancer,^[26]^ cervical cancer,^[27]^ esophageal squamous cell carcinoma^[28]^ and prostate cancer.^[29]^ The basic characteristics of the 13 articles included in the meta-analysis is listed in Table 1. The patients did not undergo any anti-cancer treatment before surgery. The articles used Kaplan-Meier curves to show the relationship between the overall survival and the level of SNHG12 expression, which was determined by qRT-PCR. Number of patients included in the analysis is no less than 30 and the follow-up period is no less than 45 months. The articles were published in English and the patients were both selected from Chinese hospitals.

**Table 1:**
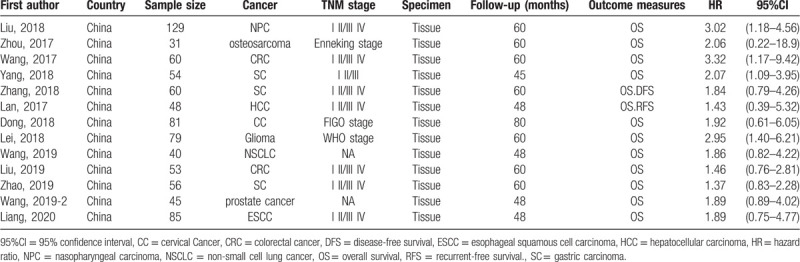
Basic characteristics of the 13 articles included in the meta-analysis of the systematic review.

### 3.3 Quality Evaluations of the included studies

We used the Newcastle-Ottawa Scale (NOS) to assess the quality of the included studies. As has shown in Table 2, 8 articles got 7 stars and 5 studies got 8 stars. All studies have more than 7 stars, which means they are up to standard to undergo meta-analysis.

**Table 2:**
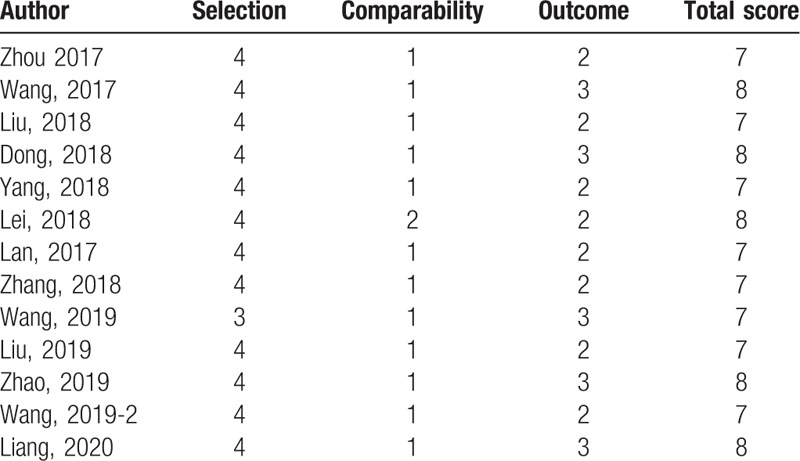
Quality assessment based on the Newcastle-Ottawa Scale (NOS).

### 3.4 The association between lncRNA SNHG12 expression and overall survival (OS)

**Figure 2:**
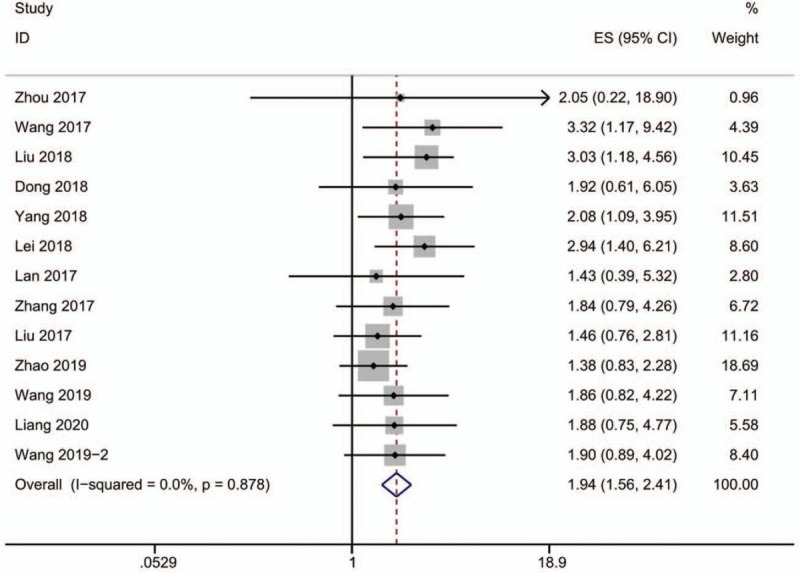
Forest plot of the relationship between SNHG12 expression level and overall survival (OS) (Fixed-effect model).

The statistics extracted from Kaplan-Meier curves in 13 articles were used to calculate HR and 95%CI. As shown in Fig. 2, the forest plot shows that the studies are lack of statistically significant heterogeneity, with the *P* value of 0.878 and *I*^2^ of 0.0%. Hence, we used the fixed-effects model to calculate the HR and 95%CI, which has been showed in Table 3(HR = 1.94, 95% CI: 1.56–2.461 *P* < .001). From what has been discussed above, we may safely draw a conclusion that patients with higher lncRNA SNHG12 expression in all types of solid malignant tumor may suffer a worse clinical outcome.

**Table 3:**
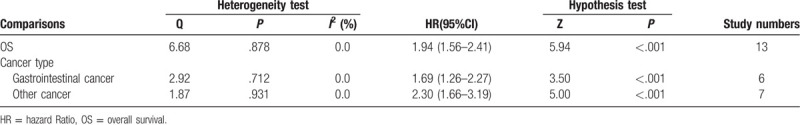
Main results of the pooled hazard ratios (HRs) in the meta-analysis.

### 3.5 Analysis of sensitivity and publication bias

**Figure 3:**
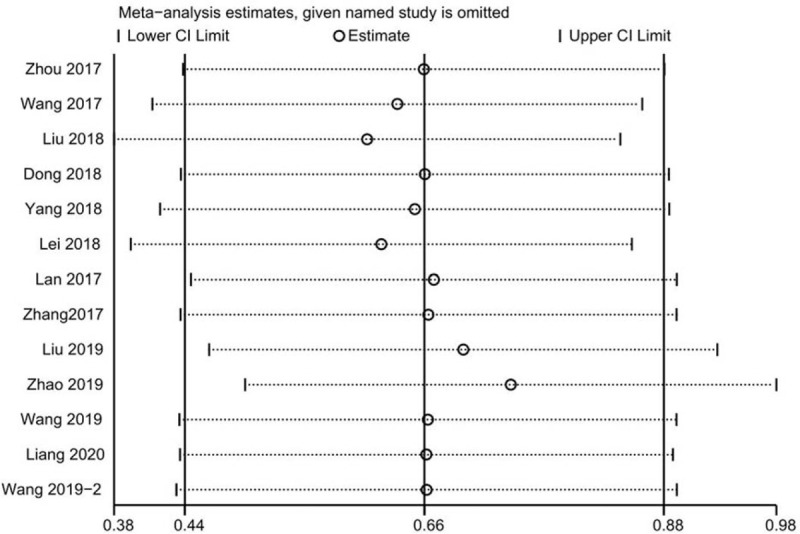
The sensitivity of the meta-analysis for overall survival (OS).

**Figure 4:**
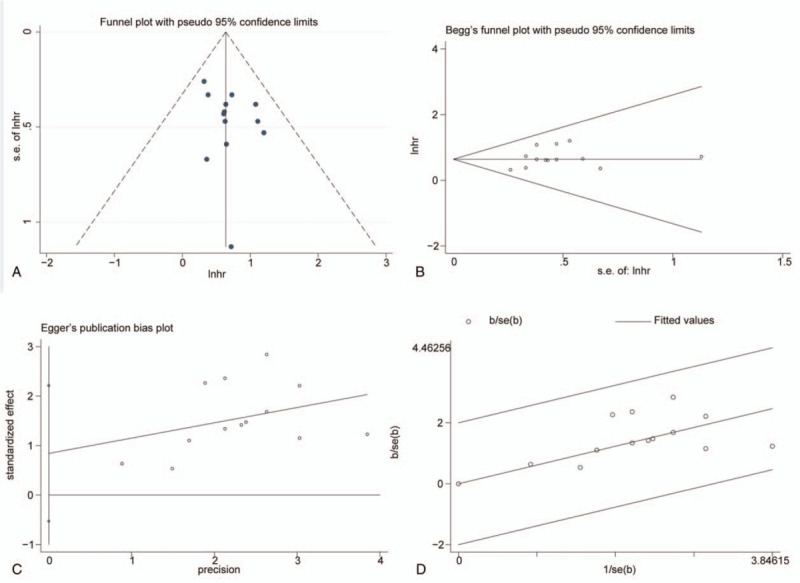
Publication bias analysis of overall survival (OS) data. (A) funnel plot (B) Beggs funnel plot (C) Eggers publication bias plot (D) Galbraiths radial plot.

Stata12 software was used to evaluate the sensitivity analysis and the publication bias so as to assess whether these studies are qualified for meta-analysis. Fig. 3 shows that the pooled HR did not change obviously when eliminate one of these studies, meaning that individual studies do not affect the pooled results. Funnel plot, Beggs funnel plot and Eggers test was used to check the existence of publication bias (Fig. 4). As shown in the figure, there is no publication bias in these studies. We used the Galbraiths radial plot to examine heterogeneity in the analysis as a supplement to the forest plot (Fig. 4).

### 3.6 Subgroup analysis

**Figure 5:**
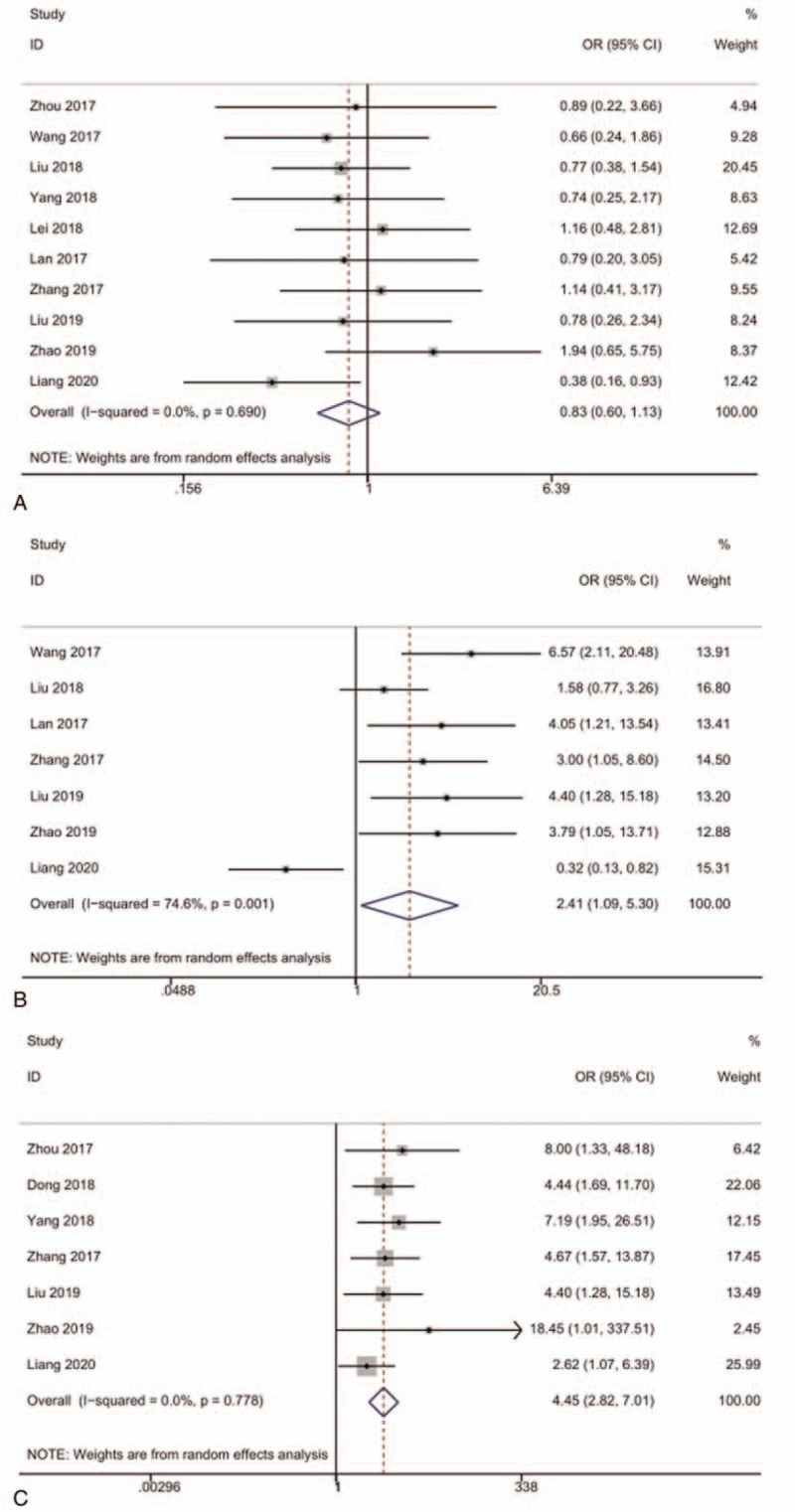
Forest plot of the relationship between SNHG12 expression and clinical characteristics. (A) gender (B) The presence of lymph node metastasis. (C) Tumor TNM stage.

**Figure 6:**
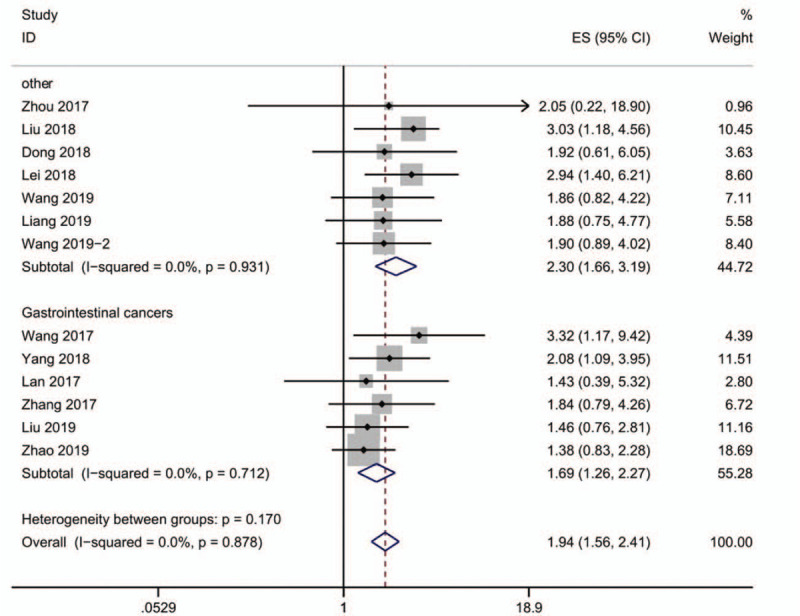
Forest plot of the relationship between SNHG12 expression and gastrointestinal cancers and other cancers (A): other cancers (B): gastrointestinal cancers. (Gastrointestinal cancers): colorectal cancer, hepatocellular carcinoma and gastric cancer. Other cancers: nasopharyngeal carcinoma, osteosarcoma, glioma, esophageal squamous cell carcinoma, non-small cell lung cancer, prostate cancer and cervical cancer).

We performed additional analysis by dividing patients into several groups, according to the classification of gender, TNM stage, lymph node metastasis (Fig. 5) and cancer type (gastrointestinal cancers vs “other cancers”) (Fig. 6). Because of the lack of the heterogeneity, a fixed-effect model was used to perform the subgroup analysis. The detailed data of the analysis is illustrated in Tables 3 and 4, from which we could draw a conclusion that there is no connection between gender and lncRNA SNHG12 expression (OR = 0.83, 95%CI, 0.60–1.13, *P* = .690, *I*^2^ = 0.0%). We discovered that lymph node metastasis and TNM stage may act as an influence factor of lncRNA SNHG12 expression, reflecting in that positive lymph node metastasis (OR = 4.45, 95%CI, 2.82–7.01, *P* = .778, *I*^2^ = 0.0%) and higher stage of TNM stage (OR = 2.41, 95%CI, 1.09–5.30, *P* = .001, *I*^2^ = 74.6%) lead to higher lncRNA SNHG12 expression. As shown in Fig. 6, the gastrointestinal cancer (HR = 1.69, 95%CI, 1.26–2.27, *P* = .712, *I*^2^ = 0.0%) shows a better outcome than other cancer (HR = 2.30, 95%CI, 1.66–3.19, *P* = 0.931, *I*^2^ = 0.0%).

**Table 4:**
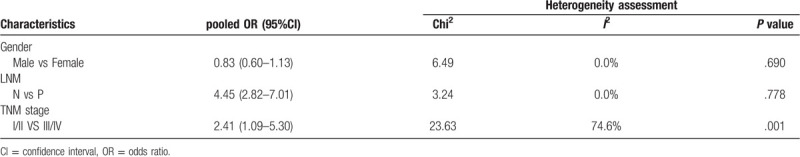
The association between low expression levels of SNHG12 and clinicopathological characteristics.

## 4 Discussion

### 4.1 Summary of evidence

The primary finding from this systematic review and meta-analysis is that patients with higher lncRNA SNHG12 expression appear to be significantly associated with worse prognosis and SNHG12 may be an unfavorable prognosis factor.

Cancer is a public health problem and an important cause of mortality worldwide, ranking the second leading cause of death in US. Evidence indicates that lncRNA (long non-coding RNA) appears to be a promising drug target in cancer therapy.^[31–33]^ LncRNA can regulate biological activities such as proliferation and metastasis in tumor cells to inhibit or induce tumorigenesis. Zhangs study^[34]^ showed that lncRNA CPS1-IT1 has a tumor-suppressive role in colorectal cancer, suggesting a function of prognosis biomarker and therapy target for lncRNA CPS1-IT1. Studies performed by Wang et al reported that lncRNA PSLNR may exert an inhibiting effect on prostate cancer via the p53-dependent pathway.^[35]^ However, lncRNA also act as an oncogene. JIN et al reported that lncRNA MALAT1 was upregulated in epithelial ovarian cancer tissues via PI3K/AKT pathway.^[36]^ HCP5, a lncRNA that regulated by TGF-β/SMAD3, plays a promotion role in lung adenocarcinoma metastasis by sponging the miR-203.^[37]^

SNHG12, an RNA gene that affiliated with the long non-coding RNA class, has been reported to be in connection with many disease occurrences, for instance, ischemic stroke. Several studies have reported that SNHG12 seem to confer a protective effect to cerebral ischemia/reperfusion injury in many pathways.^[38,39]^ By upregulating SIRT1 expression, SNHG12 alleviates cerebral ischemia/reperfusion injury through AMPK pathway.^[40]^ With the progress in the research, SNHG12 has been gradually recognized to be involved in tumorigenesis. Studies reported that lncRNA SNHG12 contribute to cell proliferation and migration by upregulating the expression of angiomotin in human osteosarcoma cells.^[41]^ Through wnt/β-catenin pathway, SNHG12 promotes cell proliferation in thyroid carcinoma^[42]^ and prostate cancer.^[43]^ MicroRNA-199a/b-5p plays a targeted role to SNHG12 in gastric cancer^[21]^ and hepatocellular cancer.^[24]^ However, the relationship between lncRNA SNHG12 expression and patients clinical outcome with solid malignant tumor is still unclear, and that's the reason for us to do this systematic review and meta-analysis.

We synthesized the essential information and survival data of 13 articles including 821 patients and 10 types of cancer. The relationship between overall survival (OS) of patients and survival time was depicted in Kaplan–Meier curves, from which we extracted data and calculated HRs, ln (HRs) and 95%CI. HRs (Hazard Ratios) represent instantaneous risk over the study time period and suffer somewhat less from selection bias, for what we choose HRs as summary measures in this meta-analysis. No heterogeneity was found in the 13 studies (*P* = .756, *I*^2^ = 0.0%), and then a fix-effects model was used. We drew a conclusion based on the meta-analysis outcome (HR = 1.94, 95% CI: 1.56–2.41, *P* < .001) that solid malignant tumor patients with high lncRNA SNHG12 expression is associated with poor survival rate, indicating that lncRNA SNHG12 may be an unfavorable prognosis factor to cancer.

We did Beggs test and Eggers test to assessed the publication bias possibility. Furthermore, some additional analyses were performed to test the existence of heterogeneity, such as funnel plot and Galbraith plot. Subgroup analyses were also performed, showing that TNM stage and lymph node metastasis may affect the expression of lncRNA SNHG12. We divided articles by cancer types and the outcome of the subgroup analysis shows that the patients suffering gastrointestinal cancer may present a better outcome compare to that suffering other cancer, and the specific reasons remains to be detected. Meanwhile, there is no significantly connection between the lncRNA SNHG12 expression and gender.

What should be highlight is that, to our knowledge, this is the first meta-analysis to explore the relationship between SNHG12 and cancer clinical outcome. Also, this meta-analysis is reported according to PRISMA statement, which makes our meta-analysis a relatively credible study.

### 4.2 Limitations

Although we did several additional analyses to perfect our study, there are still a number of limitations that should be considered while interpreting the results. Firstly, the RFS (Recurrence Free Survival) and DFS (Disease-free Survival Rates) of tumor patients were not mentioned in most articles, and that both of these studies were performed by Chinese scholars and the patients were all Chinese, which is uncapable to be a representative sample of Asian people. Secondly, the overall survival rate was extracted from the Kaplan–Meier curves using Engauge Digitizer software rather than given from the articles, leading to a possibility of deviation of the analysis. Thirdly, the cut-off value of lncRNA SNHG12 expression was not mentioned in the articles, and the subgroup analysis about age and tumor size failed to proceed, for the included literatures had different standard to grouping patients into subgroups in age and tumor size. Fourthly, the relationship of tumor histological grading and SNHG12 expression could not be tested for the lack of original data, like that carcinomas/epithelial or sarcomas/mesenchymal histogenesis were not mentioned in the included studies, which need further analysis in future study.

## 5 Conclusion

In this systematic review and meta-analysis, we found that high lncRNA SNHG12 expression is significantly associated with poor prognosis of solid malignant tumor patients. Lack of studies hindered further analysis. Hence, the relationship between SNHG12 expression in other types of solid malignant tumors should be examined in the future investigations.

## Author contributions

X-ZY managed the project and revised the draft; H-ZX and X-RQ performed literatures search and review, Z-W and H-ZX performed data extraction and meta-analysis, and write the systematic review. Database construction and data analysis was performed with the assistance of W-ZL and X-Y. All authors took part in the interpretation of the results and prepared the final version.

## Abbreviations

Abbreviations: 95%CI = 95% confidence interval, HR = hazard ratio, LncRNA = long noncoding RNA, NOS = Newcastle-Ottawa Scale, OS = overall survival, PRISMA = Preferred Reporting Items for Systematic Reviews and Meta-Analyses, SNHG12 = small nucleolar RNA host gene 12, SNHGs = small nucleolar RNA host genes.
